# Systematic Analysis Reveals Elongation Factor 2 and α-Enolase as Novel Interaction Partners of AKT2

**DOI:** 10.1371/journal.pone.0066045

**Published:** 2013-06-18

**Authors:** Katharina Bottermann, Michael Reinartz, Marian Barsoum, Sebastian Kötter, Axel Gödecke

**Affiliations:** Department of Cardiovascular Physiology, Heinrich-Heine-University Düsseldorf, Düsseldorf, Germany; University of South Florida, United States of America

## Abstract

AKT2 is one of the three isoforms of the protein kinase AKT being involved in the modulation of cellular metabolism. Since protein-protein interactions are one possibility to convey specificity in signal transduction, we performed AKT2-protein interaction analysis to elucidate their relevance for AKT2-dependent cellular functions. We identified heat shock protein 90 kDa (HSP90), Cdc37, heat shock protein 70 kDa (HSP70), 78 kDa glucose regulated protein (GRP78), tubulin, GAPDH, α-enolase and elongation factor 2 (EF2) as AKT2-interacting proteins by a combination of tandem affinity purification and mass spectrometry in HEK293T cells. Quantitative MS-analysis using stable isotope labeling by amino acids in cell culture (SILAC) revealed that only HSP90 and Cdc37 interact stably with AKT2, whereas the other proteins interact with low affinity with AKT2. The interactions of AKT2 with α-enolase and EF2 were further analyzed in order to uncover the functional relevance of these newly discovered binding partners. Despite the interaction of AKT2 and α-enolase, which was additionally validated by proximity ligation assay (PLA), no significant impact of AKT on α-enolase activity was detected in activity measurements. AKT stimulation via insulin and/or inhibition with the ATP-competitive inhibitor CCT128930 did not alter enzymatic activity of α-enolase. Interestingly, the direct interaction of AKT2 and EF2 was found to be dynamically regulated in embryonic rat cardiomyocytes. Treatment with the PI3-kinase inhibitor LY294002 before stimulation with several hormones stabilized the complex, whereas stimulation alone led to complex dissociation which was analyzed *in situ* with PLA. Taken together, these findings point to new aspects of AKT2-mediated signal transduction in protein synthesis and glucose metabolism.

## Introduction

Signal transduction by phosphorylation involves the interaction of the kinase and its substrate protein. Therefore, the chemical environment at the kinase-substrate interface is an essential determinant of substrate specificity. In higher eukaryotes protein kinases are frequently organized in kinase families resembling closely related proteins with high sequence homology [1]. A prominent example is the protein kinase B or AKT family of protein kinases which acts as a signaling node being involved in the regulation of important cellular functions such as cellular growth [2], cell cycle control [3], apoptosis [4], and metabolism [5]. AKT kinases are 57 kDa proteins which are activated by phosphorylation in response to stimulation of receptor tyrosine kinases by their specific ligands including insulin, insulin-like growth factor-1 (IGF-1), nerve growth factor-1 (NGF-1), and platelet derived growth factor (PDGF). Ligand binding to these receptors activates PI3-kinase which forms phosphatidylinositol-(3,4,5)-trisphosphate (PIP_3_) at the plasma membrane. This, in turn, triggers the translocation of the PH-domain containing AKT kinases to the cell membrane where they are activated by phosphorylation of threonine 308 and serine 473 [6] by phosphoinositide-dependent kinase 1 (PDK-1) [7] and mammalian target of rapamycin complex 2 (mTORC2) [8], respectively. After activation AKT dissociates from the plasma membrane and phosphorylates many downstream targets. There are three different AKT-isoforms (AKT1-3), which seem to regulate, at least in part, specific biological processes. AKT1 knockout mice are smaller than wild type mice suggesting a major role in cell- and body growth [9]. AKT3 affects the development of the nervous system since the brains of AKT3-knockout mice are substantially smaller than those of wild type mice [10]. AKT2 knockout mice display alterations of the metabolism characterized by a diabetes-like phenotype with reduced glucose tolerance [11]. Moreover, the insulin-induced translocation of the glucose transporter type 4 (GLUT4) to the plasma membrane depends on the increased accumulation of AKT2 but not AKT1 at the plasma membrane [12].

Systematic analysis of protein complex formation by tandem affinity purification (TAP) combined with mass spectrometry (MS) in the model organism S. cerevisiae has led to the identification of the interactome of proteins, a widespread network of stable protein complexes [13], [14]. This seminal work has clearly altered our view on protein functions which seem to be executed not only by freely diffusing proteins. They appear to be organized to a large extent in specific networks which may allow a concerted action. For AKT only a few interacting proteins are known. Well characterized is the interaction with HSP90 which not only stabilizes AKT [15], [16] but also triggers complex formation with other proteins such as Cdc37 and endothelial NO synthase (eNOS) [17].

In view of the importance of protein interactions in signal transduction we hypothesized that the systematic analysis of AKT2 associated protein complexes might result in the identification of new AKT2 interacting proteins which would give insight into novel AKT2-dependent cellular functions. Therefore, we expressed tagged AKT2 and performed an analysis of AKT2 interactions using TAP-MS/MS analysis combined with SILAC. Candidate proteins were validated with the proximity ligation assay, which also served to analyze changes of complex formation depending on AKT2 activity.

## Materials and Methods

### Ethics Statement

All procedures concerning animal handling were in accordance with the German Protection of Animals Act, executed by the local Animal Care and Use Committee and registered under permission number O/29/2011. The rats used in this study were kept at the Tierversuchsanlage Heinrich-Heine-University Düsseldorf, Germany. They were fed a standard chow diet and received water ad libitum. Rats were anesthetized with isofluran (2%) and killed by cervical dislocation.

### Vectors

To perform TAP, the AKT2-protein was N- and C-terminally fused with a TAP-Tag, consisting of HA-Tag, StrepII-Tag and FLAG-Tag. For stable expression HEK293T cells were transfected using a lentiviral vector conferring puromycin resistance allowing selection of stably transfected cells (Fig. S1).

### Cell Culture and Sample Preparation

HEK293T cells (ECACC/HPA) were cultured in Dulbecco’s Modified Eagle Medium DMEM (Invitrogen) under standard conditions. Cell lysis was performed with 150 mM NaCl, 10 mM Tris, 0,1% NP 40, pH 7,4. Roche proteinase/phosphatase-inhibitor cocktail was added immediately before lysis. Lysates were clarified by 30 minutes centrifugation (4°C, 4000 rpm).

### Cell Stimulation

HEK293T cells were treated with 25 µM LY294002 (Sigma) for one hour, 1 µg/ml IGF-1 (Milteny) or 1,75 µg/ml insulin (Sanofi Aventis) for 10 minutes. Embryonic E18 rat cardiac myocytes were serum deprived with 1% FBS supplemented medium and then treated with 50 µM LY294002 for one hour or with 100 nM angiotensin II (Sigma) for 30 minutes.

### SILAC-labeling

Cells were labeled with ^13^C_6_ Lysine and ^13^C_6_ Arginine using the *SILAC™ Protein ID & Quantitation Media Kit Lysine (DMEM-Flex)* and *SILAC™ Stable Isotopic [^13^C_6_]-L-Arginine* (Invitrogen). Medium was prepared as described by the manufacturer. Cells were fed with SILAC medium over 5 passages to enable full incorporation of the labeled amino acids.

### Tandem Affinity Purification (TAP)

Cell lysates were incubated for 1 h, 4°C, (400 rpm on a rotating platform) with Anti-FLAG M2 Affinity Gel (Sigma-Aldrich). After incubation, beads were allowed to settle and the supernatant was removed. The resuspended beads were transferred to a Micro Bio-Spin column (BioRad). Beads were washed with 10 column volumes TBS (150 mM NaCl, 3 mM KCl, 25 mM Tris, pH 7,4). Elution of the proteins was performed with 1 ml triple-FLAG peptide (N-Met-Asp-Tyr-Lys-Asp-His-Asp-Gly-Asp-Tyr-Lys-Asp-His-Asp-Ile-Asp-Tyr-Lys-Asp-Asp-Asp-Asp-Lys-C) (300 µg/ml in TBS). The FLAG eluate was then affinity purified on a Strep-Tactin Sepharose (IBA Technologies) column. Beads were washed with 10 column volumes Strep buffer (100 mM Tris, 150 mM NaCl, 1 mM EDTA, pH 8,0). Tagged proteins were eluted with 2,5 mM Biotin (Sigma-Aldrich) in Strep buffer. The Strep-eluate was concentrated with Centrifugal Filter Units, 10K Membrane (Millipore).

### Silver Staining and Image Analysis

MS compatible silver staining was performed according to Shevchenko et al. [18] with minor modifications as described before [19].

### Tryptic Digestion

In gel digestion was performed as described [19]. To digest TAP purified proteins in solution 8 M urea was added to the concentrated sample. Proteins were reduced with 10 mM DTT for 1 h at 30°C and then alkylated with 40 mM iodoacetamide for 1 h at 30°C. Afterwards the solution was diluted 1∶10 in 50 mM NH_4_HCO_3_, 5 mM Ca_2_Cl, concentrated over Centrifugal Filter Units (10K Membrane, Millipore) and digested with trypsin for 16 h at 37°C.

### Nano-LC-MS/MS Analysis

Proteolytic digests were analyzed with an LTQ-Orbitrap XL mass spectrometer (Thermo Scientific) after separation by nano-LC as described earlier [19]. To conduct peptide analysis after in solution digest peptide mixtures were subjected to automated off-line 2-D LC-MS/MS using the microfraction collection option of the WPS 3000PL autosampler. Peptides were separated on a 1 mm×15 cm Polysulfoethyl-Aspartamide column (Dionex, Thermo Scientific) in the first dimension by gradient elution from 0–60% B for 30 min, 60–100% B for 5 min (A: 5 mM NaH_2_PO_4_, pH 2.7; B: 5 mM NaH_2_PO_4_, 15% acetonitrile, 500 mM NaCl, pH 2.7) at a flow rate of 50 µl/min. One minute fractions collected by the autosampler were re-injected for reverse-phase nano-LC-MS/MS. Each MS full scan (m/z 350–2000, acquired at a target value of 1,000,000 ions with resolution r = 60,000 at m/z 400) was followed by MS/MS scans of the ten most intense ions, whereby the normalized collision energy for collision induced dissociation was set at 35 units, and a repeat count of 1 with a 20 sec exclusion duration window was applied (exclusion mass width of 10 ppm). Multistage activation was enabled upon detection of a neutral loss of phosphoric acid for further fragmentation.

### Sequence Database Searching, Protein Identification and Relative Quantification

Sequence database-search of the MS data was performed against the uniprot human taxonomy-9606 database containing 83659 entries using the SEQUEST algorithm with the following parameters: trypsin specificity, two missed cleavage sites, precurser ion mass accuracy tolerance of 10–30 ppm, cysteine carbamidomethylation, methionine oxidation, pSTY, N-terminal protein acetylation and, when performed, SILAC labels Lys-6, Arg-6 specified as modifications. The minimal cross-correlation score (XCorr) was set to 2.0, 2.5 and 3.0 for charge states +2, +3 and +4 respectively. The ΔCN had to be >0.1 and the minimal peptide probability allowed was 0.05. The minimum number of peptides necessary for protein identification was three. Relative quantification of peptides/proteins was carried out with the PepQuan feature of the Bioworks Browser 3.3.1 SP1 (Thermo Fisher Scientific). Peak areas were calculated with a mass tolerance of 0.1, a minimum threshold of 15000 and no smoothing. When necessary, peak integration was manually corrected. Each protein ratio was calculated as mean value from a minimum of three unique peptide ratios. If a peptide ratio could not be calculated due to the absence of the corresponding peptide in the control sample, the ratio was set as ∞. Accordingly, the corresponding protein ratio was also ∞.

The mass spectrometry proteomics data of the SILAC-TAPs have been deposited to the ProteomeXchange Consortium (http://proteomecentral.proteomexchange.org) via the PRIDE (PRoteomics IDEntifications Database) partner repository [20] (http://www.ebi.ac.uk/pride/) with the dataset identifier PXD000197 and DOI 10.6019/PXD000197. (PRIDE accessions MBP-TAP I: 29063–29102, MBP-TAP II: 29103–29151, MAP-TAP I: 29152–29201, MAP-TAP II: 29202–29251).

### Western Analysis

Proteins were transferred to nitrocellulose membranes (Whatman, Schleicher & Schuell) after SDS-PAGE as described before [21]. All primary antibody incubations were performed in Tris-buffered saline (137 mM NaCl, 20 mM Tris, pH 7,6) with 0,1% Tween 20 containing 5% BSA at 4°C overnight using dilutions as recommended by the manufacturer. After incubation with primary antibodies, blots were washed three times for 10 minutes. Incubations with secondary antibodies were performed at a dilution of 1∶15000 in Odyssey Blocking Buffer (LI-COR) at room temperature for 1 h. After washing (three times for five minutes), the blots were developed with the LI-COR Odyssey® Infrared Imaging System and the Odyssey Software V2.1 or V3.0. Primary Antibodys: AKT-Antibody (9272), AKT Mouse mAb (2966), AKT2 Rabbit mAb (3063), AKT2 Mouse mAB (5239), HA-Tag Mouse mAB (2367), p-AKT (Ser 473) Antibody (9271), p-AKT (Thr 308) Antibody (9275), p-GSK3β (Ser9) Antibody (9336), eEF2-Antibody (2332), GAPDH Rabbit mAb (2118) (all Cell Signaling Technology), anti-ENO1 antibody (abcam). Secondary Antibodys: IRDye 800CW Goat Anti-Mouse IgG, IRDye 800CW Goat Anti-Rabbit IgG, IRDye 680 Goat Anti-Mouse IgG, IRDye 680 Goat Anti-Mouse IgG (LICOR).

### Proximity Ligation Assay

Cells were plated on adhesive microscope slides with silane treated surfaces (Marienfeld) in 20 mm^2^ areas, marked with a liquid-repellent slide marker pen (Daido Sangyo Co., Ltd.). Areas were covered with 0,1% gelatine before cell plating. Cells were allowed to settle down for 4 h or overnight, washed in PBS (137 mM NaCl, 2,7 mM KCl, 8,1 mM Na_2_HPO_4_× 2H_2_O, 1,76 mM KH_2_PO_4_, pH 7,4), fixed with 4% paraformaldehyde in 0,1 M sodium phosphate buffer pH 7,4 and permeabilized with 0,2% saponin in PBS. Primary antibodies were used in appropriate concentration (1∶100–1∶500) and incubated overnight at 4°C. Afterwards the PLA-protocol was performed with Duolink InSitu reagents (Olink Bioscience) as described before [22]. Slides were mounted with Duolink InSitu Mounting Medium with DAPI. Primary Antibodies (Cell Signaling Technology, abcam): AKT2 Mouse mAB (5239), eEF2-Antibody (2332), GAPDH Rabbit mAB (2118), AKT1 Mouse mAB (2967), anti-ENO1-antibody (ab85086). Slides were examined under the fluorescence microscope Keyence BZ 9000 (Keyence) using a 60× objective. To evaluate protein interactions, five to ten image sections were analyzed for the ratio of signals per nuclei. The diagrams show relative numbers of signals per nuclei. The PLA-samples were set to 100%. The signals/nuclei of the corresponding controls are shown as percentage of full PLA for each experiment.

### ENO1 Activity Assay

To measure activity of α-enolase, the ENO1 Human Activity Assay Kit (Abcam, ab117994) was used. α-enolase was purified from cell lysates by immuno-affinity purification and then incubated with a solution which contained the substrate of α-enolase, 2-phosphoglycerate, which was converted to phosphoenol pyruvate (PEP). PEP was then further processed by pyruvate kinase to pyruvate which is used by lactate dehydrogenase to form lactate under NADH-consumption. The decrease of NADH-concentration was measured as change in NADH-absorbance at 340 nm. HEK293T wild type cells were stimulated with insulin (1,75 µg/ml) for 10 minutes or treated with 10 µM CCT128930 (AKT-inhibitor) (SelleckBio.com) [23] for 30 minutes. Sample preparation and assay procedure was performed as described by the manufacturer using a protein concentration of 10 µg/ml. NADH-absorbance was measured at 25°C with a Fluostar Optima plate reader (BMG Labtech) at 340 nm.

### Preparation of Embryonic Rat Cardiomyocytes (E18)

Adult pregnant Wistar rats were anesthetized with isofluran (2%) and killed by cervical dislocation. Embryos were decapitated before preparing the hearts. All procedures were in accordance with the guidelines of the local Animal Care and Use Committee. Cardiomyocytes were isolated by enzymatic dissociation with collagenase Type II (Gibco, 1 mg/ml) and trypsin (Gibco, 3 mg/ml) in dissociation buffer and plated at a density of 5×10^5^ cells/well and cultured at 37°C and 5% CO_2_ in Dulbecco’s Modified Eagle Medium (DMEM). For further information see online supplement (Protocol S1). On the next day cells were treated with mitomycin (Sigma, 10 µg/ml) for 1 hour to prevent further proliferation of fibroblasts.

## Results

### TAP-MS Analysis of AKT2-interacting Proteins

**Figure 1:**
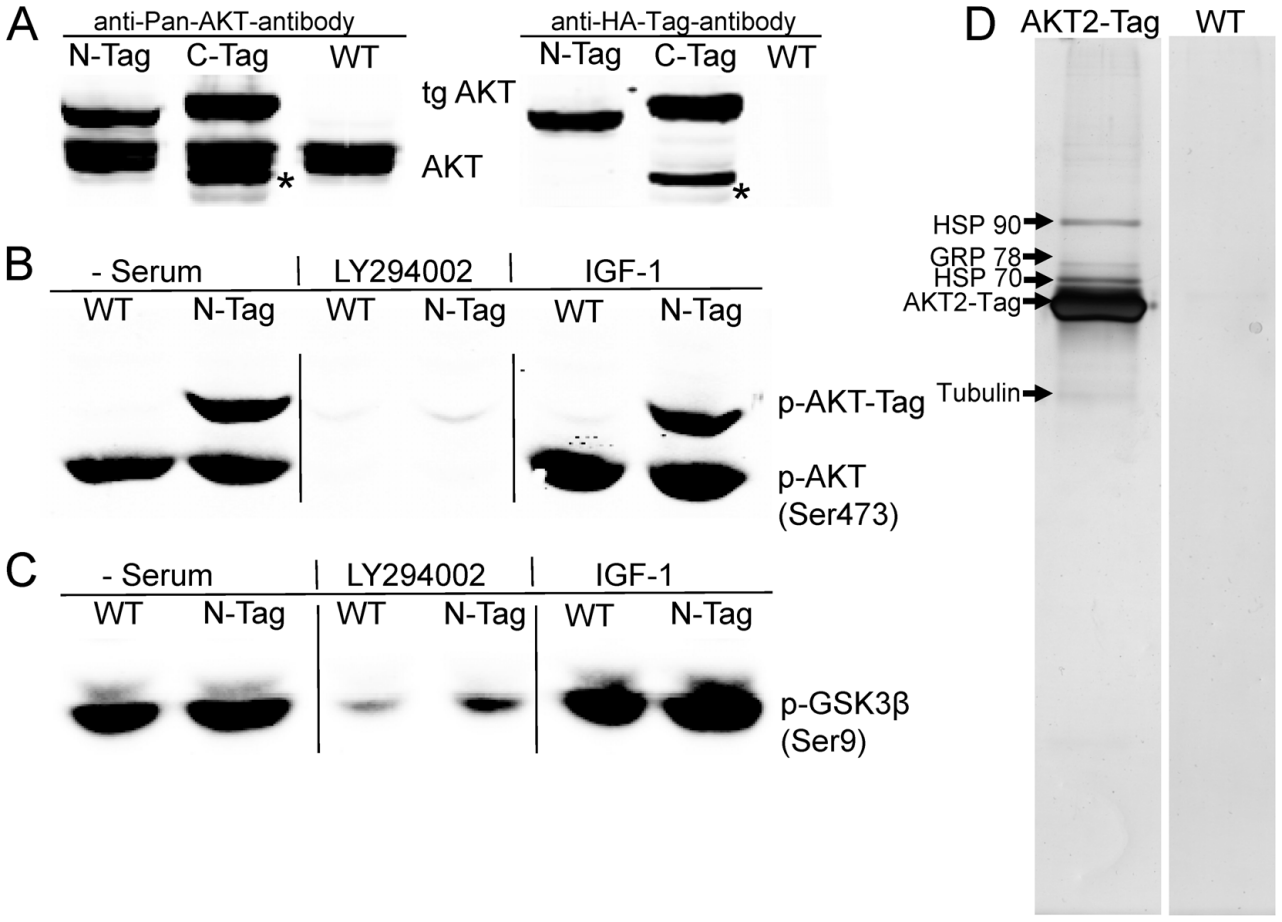
Characterization of tagged AKT2. A: Expression of C- and N-terminally tagged AKT in comparison to endogenous AKT. Tagged AKT was stably expressed in HEK293T cells and detected by pan-AKT and anti-HA-tag antibodies. *degradation product of C-terminally tagged AKT at 50 kDa, detected by pan-AKT-antibody as well as HA-Tag antibody. B: Regulation of serine 473 phosphorylation in AKT2-NTag. Stably AKT2-NTag expressing cells and WT cells were serum deprived overnight, treated with LY294002 (1 h, 25 µM) or stimulated with IGF-1 (10 minutes, 1 µg/ml). C: Regulation of AKT substrate GSK3β phosphorylation at serine 9. D: Silver stained SDS-gel of proteins isolated from AKT2-NTag and wild type cells (WT) by TAP.

To investigate protein binding to AKT2 we fused the AKT2 coding sequence with a short nucleotide sequence encoding the HA-, FLAG-, and StrepII-tags all of them suitable for affinity purification of the tagged protein and native elution from the affinity columns by either competing peptides (HA, FLAG) or biotin (Strep) (Fig. S1). Both, N- and C-terminally tagged AKT2 proteins were constructed and expressed in HEK293T cells. As shown in Fig. 1A lentiviral transfection of HEK293T cells resulted in expression levels of both proteins which were similar to the amount of endogenous AKT. However, Western analysis also revealed protein degradation in case of the C-Tag. We therefore performed all our experiments with cells, stably expressing N-tagged AKT2 which was expressed at a ratio of approximately 2∶1 and 1∶2 in comparison to the endogenous AKT2 and total endogenous AKT, respectively. Physiological regulation of tagged AKT2 was confirmed by stimulation experiments with IGF-1 or PI3-kinase inhibition with LY294002 (Fig. 1B). The efficiency of AKT inhibition was assessed by determination of the glycogen synthase kinase 3β (GSK3β) phosphorylation level of serine 9 which is a target for AKT-dependent phosphorylation (Fig. 1C).

AKT2-Tag protein and bound interaction partners were purified via anti-FLAG M2 and Strep-Tactin Sepharose beads. The purification procedure resulted in a recovery of ∼18% of AKT2-Tag protein (Fig. S2). The analysis of six independent TAP experiments by separation of the purified proteins via SDS-PAGE and silver staining uncovered a straightforward, quite reproducible pattern of candidate proteins which were copurified with AKT2, while none of them could be detected in the WT control (Fig. 1D). As summarized in Table 1 known AKT-binding proteins and also several new candidates emerged from these TAP experiments (a detailed list of identified proteins is given in Table S1). Among the identified proteins the known AKT-interaction partners HSP90 and Cdc37 were highly abundant and detected in all TAP experiments. As the HSP90/Cdc37 complex promotes AKT-stability and substrate scaffolding, these findings were important “positive controls” for the reliability of the TAP-MS approach. In addition to these very important interaction partners, we also found chaperones of the heat shock protein 70 kDa (HSP70)-family like HSP70 or GRP78. The chaperones represented the most prominent bands next to AKT in the gel after silver staining. Moreover, we also identified proteins from rather faint bands as GAPDH, EF1α, Tubulin, EF2 and α-enolase. Being slightly above the detection limit, these proteins did not appear in all TAPs.

**Table 1:** Overview of proteins most frequently detected in six (I-VI) TAPs with AKT2-NTag.

TAPs n = 6	found n =	amino acid sequence coverage (%)						MW (kDa)
*TAP #*		*I*	*II*	*III*	*IV*	*V*	*VI*	
**Proteins**								
Heat shock protein 90 kDa	6	9,3	5,7	14,8	19,9	27,4	28,4	84
Cdc 37	5	x	23,3	26,2	25,1	17,7	25,7	44
Heat shock protein 70 kDa	6	6,2	5,8	5,9	16,4	24,6	18,3	70
78 kDa glucose regulated protein	5	36,5	x	39	39,1	43,3	40,2	72
Elongation factor 1α	4	x	17,3	30,5	x	26	32,2	50
Glyceraldehyde-3-phosphate-dehydrogenase	4	x	12,8	22,1	x	49,6	51,3	36
Tubulin	4	20,6	31	44,8	18	x	x	50
Elongation factor 2	3	x	x	15	x	46,4	51,4	95
α-enolase	3	x	x	35,7	x	61,3	51,1	47
Endoplasmin	3	x	x	5,2	x	32,8	38,6	92
Protein arginine N-methyltransferase 5	3	x	x	4,9	x	24,5	19,3	72

Listed are those proteins, which were identified in three to six experiments.

x: Protein was not detected.

Table S1 shows a detailed table with all identified proteins of these six TAPs.

### SILAC Experiments Uncover the Transient Nature of most AKT2-interactions

**Figure 2:**
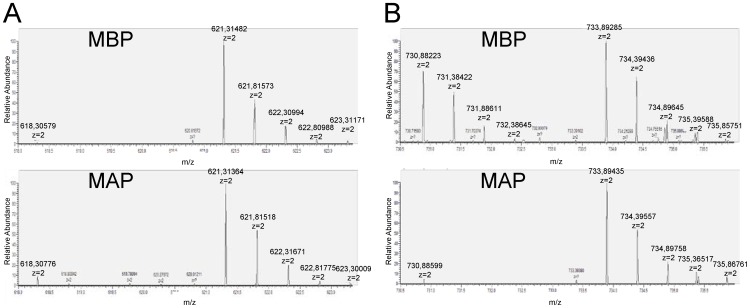
Detail of representative MS-spectra for peptide ratios of a stably and a transiently bound protein after MBP and MAP. A: Light and heavy form of the peptide DAGTIAGLNVMR of HSP90α with a shifted ratio for both approaches. B: Light and heavy form of the peptide SDIDEIVLVGGSTR of GRP78 with a ∼1∶1 ratio after MBP and a shifted ratio after MAP.

The faint bands coeluting with AKT2 from the second affinity column suggested, that AKT2 might form rather unstable protein interactions that would lead to some loss of interacting proteins during the purification procedure. To characterize the stability of protein complexes we performed TAP experiments combined with quantitative MS [24] after stable isotope labeling by amino acids in cell culture (SILAC) [25]. To this end, cells expressing tagged AKT2 were labeled with ^13^C_6_-L-arginine and ^13^C_6_-L-lysine, whereas control cells were cultured in normal medium containing light (^12^C_6_) amino acids. To investigate if AKT2 formed high affinity protein interactions, cell lysates of heavy labeled AKT2-Tag cells and unlabeled control cells were mixed in a protein ratio of 1∶1, either before (Mixing Before Purification) or after (Mixing After Purification) tandem affinity purification [26], [27], [24] (Fig. S3). Proteins forming a stable complex with AKT2 were expected to appear at a high ratio of heavy to light peptides in the MS analysis in both setups, since a stable interaction should sustain the purification procedure and an exchange with the corresponding protein from the unlabeled control sample would be negligible. For unspecific and low affinity binding proteins a ratio around 1∶1 was expected in MBP-TAPs, because the initially bound interacting proteins (^13^C_6_-labeled) may exchange against the corresponding light proteins from the wild type control during the TAP procedure. However, the ratio of heavy:light should be shifted towards the heavy form for transiently interacting proteins in MAP because no exchange with the light form of the protein can occur during TAP under these conditions. Fig. 2 shows representative MS spectra for peptide ratios of stably (HSP90) and transiently interacting proteins (GRP78) as determined by MBP and MAP. Whereas for HSP90 both, MBP- and MAP-TAP demonstrate a high ratio of heavy:light peptides, GRP78 appears to be exchanged during the MBP-purification procedure since substantial amounts of light peptides are copurified in comparison to MAP-TAP. Table 2 summarizes the results of two MAP-TAPs and two MBP-TAPs with the corresponding SILAC ratios. For HSP90 the ratio of light to heavy peptides ranged from ∼1∶22 to ∞ characterizing HSP90 as stably bound to AKT2. While the same was true for Cdc37 other identified interacting proteins (GRP78, tubulin) seem to interact only transiently with AKT2. After MBP-TAP the heavy:light ratios approximate 1∶1, whereas MAP-TAP ratios were clearly shifted towards the heavy forms. The AKT2/HSP70-interaction partially sustained the MBP-procedure (ratio ∼2∶1–5∶1) and also showed clearly shifted MAP-ratios (∼33∶1–∞) pointing to a semi stable interaction. During the SILAC-TAP experiments also nonspecific proteins were identified which most likely represented contaminants with a heavy:light ratio of 1∶1 in MAP- and MBP-experiments (PRMT5, nucleolin, methylosome protein 50) [28].

**Table 2:** SILAC-ratios of proteins found after MBP- and MAP-TAPs.

SILAC-TAPs AKT2-NTag :WT	SILAC Ratio NTag:WT				MW (kDa)
	MAP I	MAP II	MBP I	MBP II	
Heat Shock Protein 90 kDa					
Heat shock protein HSP 90-alpha	69.1±27.7	∞	∞	∞	84
Putative heat shock protein HSP 90-beta 2	80.0±39.4	∞	75.1±38.0	21.9±1.0	44
Putative heat shock protein HSP 90-beta-3	98.7±49.4	72.1±39.8	62.3±39.0	33.8±3.6*	68
Cdc 37	89.9±42.3	x	45.4±14.7	∞*	44
60 kDa heat shock protein, mitochondrial	16.9±4.5	33.6±1.6	7.4±2.7	2.3±1.4	61
Heat Shock Protein 70 kDa					
Heat shock 70 kDa protein 1	40.1±15.4	∞	3.2±0.5	3.8±0.6	70
Heat shock cognate 71 kDa protein	33.3±12.0	∞	2.6±0.4	3.1±0.4	70
Heat shock 70 kDa protein 1L	∞	∞	3.1±1.5	4.9±0.7	70
Heat shock-related 70 kDa protein 2	∞	∞	2.7±0.2	2.3±1.0*	70
78 kDa glucose regulated protein	26.0±11.6	32.2±18.2	1.5±0.8	1.0±0.4	73
Stress 70 protein, mitochondrial	x	∞	1.2±0.1	0.9±0.2	50
Tubulin alpha 1A chain	∞	∞	1.2±0.5	1.2±0.5	50

MAP: Mixing after purification, MBP: Mixing before purification.

SILAC ratios were calculated as means ± SD from a minimum of three different peptide ratios.

x: Protein was not detected.

n = 2 biological replicates.

∞: corresponding peptide from wild type sample was not detected.

* : These ratios are based on two unique peptide identifications.

Other interesting AKT2 binding proteins which were found in the initial TAP experiments including EF2, GAPDH and *α-*enolase could not be identified and thereby characterized in the SILAC experiments. Since these proteins were only slightly above the detection limit in the gel-based experiments, peptide detection might have escaped from the identification due to the presence of more abundant peptides coeluting during LC-MS/MS analysis or due to loss because of low stability of AKT interaction. Therefore, further investigation of these potential interactions by a complementary assay was performed.

### Analysis of Glycolytic Enzymes as AKT2 Interacting Partners

**Figure 3:**
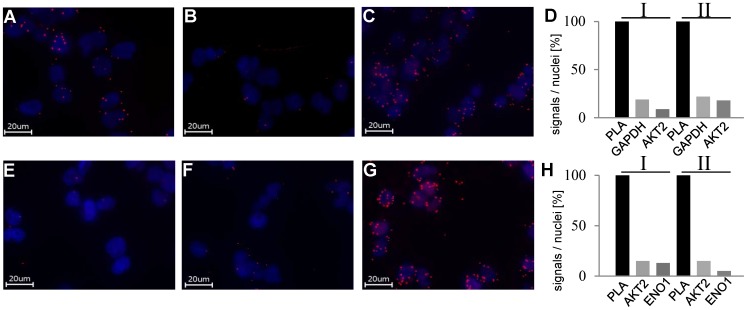
PLA results for AKT2/GAPDH (A–D) and AKT2/α-enolase (ENO1) (E–H) in HEK293T AKT2-NTag cells. A/B, E/F: Control experiments with only one of the primary antibodies used. C and G show complete PLAs. D/H: Quantitative analysis of two independent experiments for each antibody combination. The number of signals/nuclei in the PLA-sample was set as 100%. Controls are shown as percentage of full PLA.

To validate interacting proteins we used the proximity ligation assay which detects protein interactions in cells in situ. The assay detects proteins which are in close proximity (<40 nm) via antibodies and marks the interaction by a fluorescence signal [22]. We performed PLA on HEK293T AKT2-NTag cells, using an anti-AKT2 (mouse)-AB, an anti-GAPDH (rabbit)-AB and an anti-Enolase-(rabbit)-AB (Fig. 3). In the control experiments performed with one of the primary antibodies, only rare background signals for anti-AKT2- and anti-GAPDH-antibodies were observed (Fig. 3A, B). However, the combination of both antibodies revealed a high signal level which substantially exceeded that of both background signals (Fig. 3C). The quantitative analysis of the data for two independent PLA experiments in Fig. 3D supports our TAP results confirming that GAPDH interacts with AKT2 in HEK293T cells in situ. The same approach was used for the evaluation of α-enolase (Fig. 3 E–H). These experiments yielded similar results, confirming a specific interaction of AKT2 with α-enolase. In combination with the results of the TAP-experiments, we conclude, that these proteins most likely interact transiently with AKT2.

**Figure 4:**
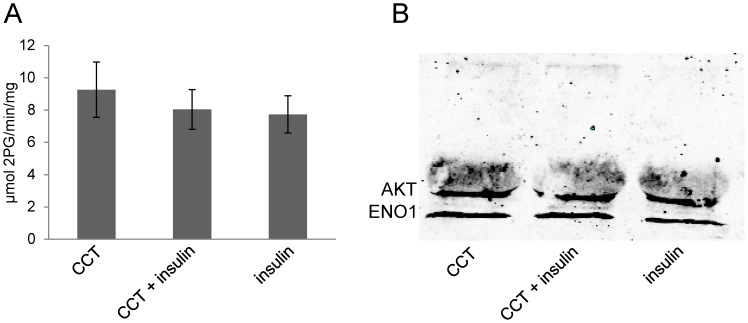
α-enolase activity assay. A: Activity of immuno-captured α-enolase (ENO1) from HEK293T cells after stimulation with insulin and AKT-inhibition with CCT128930. Data represent means ± SD of n = 7 experiments. B: Detection of immuno-captured α-enolase after α-enolase activity assay. After detection of α-enolase blots were incubated with anti-pan AKT antibody. Note that AKT was also detected after isolation of α-enolase by the α-enolase antibody.

After identification and validation of α-enolase as an AKT2-binding partner, we aimed at investigating if AKT has an influence on α-enolase activity. Therefore, we used a Human ENO1 activity assay which allows to measure α-enolase activity on protein immunocaptured from cell lysates. To analyze the influence of AKT on α-enolase activity, cells were stimulated with insulin or treated with the AKT-inhibitor CCT128930 followed by addition of insulin. CCT128930 is an ATP-competitive AKT-inhibitor, leading to inactivation of phosphorylated AKT and subsequent dephosphorylation of AKT substrates (Fig. S4). As shown in Fig. 4A insulin dependent stimulation of AKT2 and inhibition of AKT2 in insulin stimulated cells did not reveal differences in α-enolase activity. Also, cells treated only with CCT128930 showed no significant alteration in α-enolase activity. As shown in Fig. 4B similar activity was paralleled by similar amounts of α-enolase captured by the ENO1 antibodies. In order to test, if AKT still associated with the captured α-enolase, we analyzed the presence of AKT in the immunocaptured α-enolase preparations. Fig. 4B reveals that substantial amounts of AKT were copurified with α-enolase.

### AKT2/EF2 Interaction is Regulated by Several AKT-activating Stimuli

**Figure 5:**
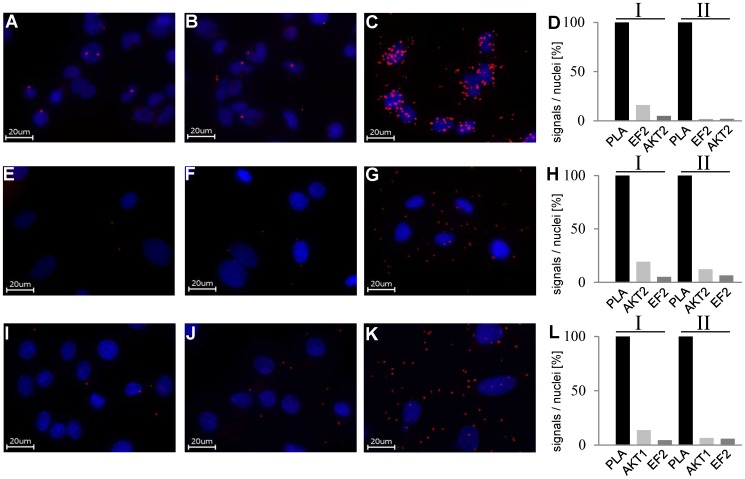
Results of PLA of AKT and EF2. A–D: PLA on HEK293T AKT2-NTag cells A/B: Control experiments with one primary antibody. C: Complete PLA. D: Quantitative analysis of two independent experiments. E–L: Results of PLA with AKT2 and AKT1 with EF2 in embryonic rat cardiomyocytes. E–H: PLA AKT2/EF2 E/F: Control experiments with one primary antibody. G: Complete PLA. H: Quantitative analysis. I–L: PLA AKT1/EF2 I/J: Control experiments with one primary antibody. K: Complete PLA. L: Quantitative analysis. The number of signals/nuclei in the PLA-samples was set as 100%. Controls are shown as percentage of corresponding full PLA.

TAP-experiments revealed also elongation factor 2 as a specific interaction partner of AKT2. To verify this interaction by a complementary approach, we performed PLA in HEK293T AKT2-NTag cells. The results, shown in Fig. 5 A–D, confirm the interaction of AKT2 and EF2.

For a closer look on functional relevance and isoform specificity of the AKT2/EF2-interaction further experiments were performed with embryonic (E18) rat cardiac myocytes. As shown in Fig. 5 E–H PLA revealed that AKT2 also interacts with EF2 in primary cardiac cells. We also addressed the question if this interaction was specific for AKT2. Fig. 5 I–L shows that AKT1 was able to interact with EF2 in embryonic rat cardiac myocytes.

**Figure 6:**
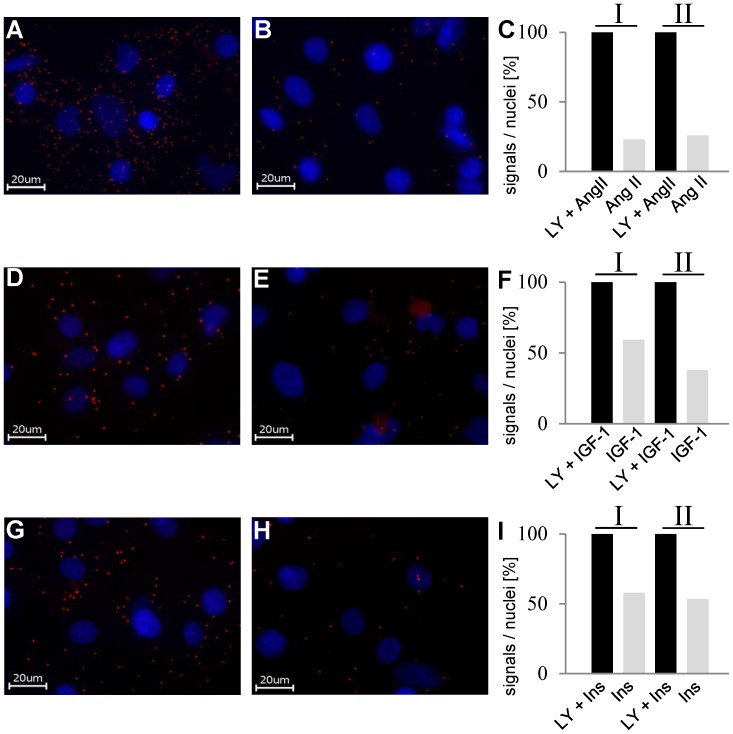
Dynamic regulation of the AKT2/EF2-interaction in embryonic rat cardiomyocytes. A/D/G: PLA after inhibition of PI3-Kinase with LY294002 (1 h, 50 µM) and subsequent angiotensin II (A), IGF-1(D) and insulin (G) stimulation B/E/H: PLA after stimulation of the cells with angiotensin II (30 minutes, 100 nM) (B), IGF-1(10 minutes, 1 µg/ml) (E) and insulin (10 minutes, 1,75 µg/ml) (H). C/F/I: Quantitative data of two independent PLA-experiments for each stimulant. The number of signals/nuclei for the LY294002+ stimulant sample was set as 100%.

As EF2 is a target for regulation of protein synthesis and AKT is known to be involved in cardiac hypertrophy, we examined the influence of several hypertrophic stimuli on AKT2/EF2-interaction. Rat cardiac myocytes were serum deprived overnight and then stimulated with angiotensin II, IGF-1, or insulin. PLA-signals obtained for these cells were compared with cells which were pretreated with the PI3-kinase inhibitor LY294002. Treatment with angiotensin II (Fig. 6 A–C), IGF-1 (Fig. 6 D–F) and insulin (Fig. 6 G–I) resulted in a reduction of PLA signal intensity to 25%–50% of that obtained for cells inhibited before by addition of LY294002. These results strongly suggest that AKT2 and EF2 mainly interact when AKT2 is inactive. After phosphorylation and activation of AKT2, the interaction falls apart.

## Discussion

The protein kinase AKT integrates a large number of cellular processes by acting as a signal diverter in most mammalian cells. Representing such an important signaling node, AKT is also involved in pathological processes, including cancer, diabetes and cardiac hypertrophy. Thus, gaining a comprehensive picture of AKT-mediated signaling is necessary, particularly for the development of new therapeutic strategies. As the systematic analysis of protein complex formation may result in discovery of new cellular functions we performed a tandem affinity purification based approach and identified new protein interactions of the AKT2-isoform.

The major findings reported in this paper are as follows:

1. TAP in combination with SILAC revealed that AKT2 dependent signaling involves rather unstable protein complexes with the exception of HSP90 and Cdc37.
2. α-enolase and elongation factor 2 (EF2) represent novel AKT2 interacting proteins.
3. The interaction of AKT2 and EF2 is reduced upon stimulation of AKT2 via insulin, IGF-1 and angiotensin II, respectively.

The modified TAP-Tag fused to the N-terminus of AKT2 allowed for proper regulation of the recombinant enzyme, which was expressed slightly higher than the endogenous isoform (ratio 2∶1). This expression level enabled the isolation of AKT2 with interacting proteins and their subsequent identification by mass spectrometry. The purification efficiency (18%) of our TAP protocol was comparable to other TAP-Tags [29]. PAGE separation followed by MS analysis of co-purified proteins demonstrated that AKT2 interacts predominantly with heat shock proteins which were previously shown to have concrete functions in the context of AKT signaling. The interaction with HSP90/Cdc37 is known to promote AKT-stability [15] and activity [16]. HSP90 and Cdc37 act as scaffold proteins which form a complex with AKT. This ternary complex appears to form the interface for the interaction with multiple AKT substrates like eNOS [17] or apoptosis signal-regulating kinase 1 (ASK1) [30]. Therefore, it is not surprising that HSP90 and, to a lesser extent, Cdc37 represented the most abundant AKT-interacting proteins. Since this interaction reproducibly sustained two affinity purification steps without appreciable interchange as demonstrated by SILAC experiments, we assume that AKT2 and HSP90 form a stable complex. Different members of the HSP70 family were also reproducibly detected. These interactions are rather stable, since the SILAC analysis revealed no substantial exchange of the AKT-associated HSP70 proteins during the TAP procedure. HSP70 proteins are also known to regulate AKT stability and degradation [31]. In addition to these stable and semi stable complexes we discovered that AKT2 rather loosely interacts with other proteins. As a further chaperone, we identified GRP78 to interact with AKT2 in a transient manner. GRP78, a classical ER chaperone which serves as a switch in the unfolded protein response, was found to be involved in several pathophysiological processes, mediated in part via the AKT pathway. It was suggested that decreased GRP78 expression may lead to insulin resistance by inhibiting AKT activation [32]. On the other hand AKT seems to regulate GRP78 expression downstream of IGF-signaling [33]. Several cancer cell lines express GRP78 on their cell surface, where it acts as a receptor which can mediate prosurvival signaling by activation of AKT [34]. In our SILAC experiments we clearly characterized GRP78 as a protein transiently interacting with AKT2.

The AKT2 interaction with GAPDH, α-enolase and EF2 could not be characterized via SILAC as their protein amounts were only slightly above the detection level of silver staining, implicating either a low cellular abundance of the complexes or a loose interaction, which does not sustain the purification procedure. We confirmed these interactions *in situ* using the proximity ligation assay.

Interaction of AKT2 with GAPDH provides a direct link to a possible contribution in the regulation of glycolysis. Two groups showed an interaction between AKT and GAPDH via immunoprecipitation [35], [36]. Besides its central role in glycolysis several other functions of GAPDH have been described. The involvement in the regulation of cell death and carcinogenesis [37] is of particular interest in the context of our finding. It has been reported that AKT2 phosphorylates GAPDH on threonine 273 and thereby exerts an antiapoptotic effect [36]. Thus, the direct interaction of AKT2 and GAPDH might facilitate the regulation of glycolysis and apoptosis by AKT.

α-enolase, another glycolytic enzyme catalyzing the conversion of 2-phosphoglycerate to phosphoenolpyruvate, was also found to interact with AKT2. We also investigated by Strep-Tactin precipitation if AKT1 interacts with α-enolase. However, a clear result could not be obtained which suggests that, if at all, AKT1 forms only a quite unstable interaction with α-enolase. Thus, α-enolase is a newly discovered AKT2-interaction partner. α-enolase activity and complex formation after stimulation and inhibition of AKT, respectively, were not affected by the different activation states of AKT. Furthermore, search for possible AKT-consensus phosphorylation sites in α-enolase using the software “scansite motif search” (http://scansite3.mit.edu/) did not reveal any hits, even under low stringency search conditions. These findings suggest that there has to be another physiological relevance of the interaction, than modulating the activity of the enzyme. A more indirect link between these two proteins can be deduced in context of the Warburg-effect. The upregulation of α-enolase in cancer cells [38] mainly occurs via its hypoxia sensitive element, which enables HIF1 (hypoxia inducible factor) to promote transcription and translation [39]. Together with upregulation of other glycolytic enzymes, tumor cells gain an increased flux through glycolysis in glucose metabolism and produce more ATP directly from pyruvate ( = Warburg-effect) [40], [41], [42]. AKT is involved in this pathway in several ways [43]. It can promote membrane integration of glucose transporters, influence the activity of the glycolytic enzymes hexokinase [44] and phosphofructokinase [45], and raise the HIF1-protein level in the cell via mTOR [46]. Our data, which revealed a direct interaction between AKT and α-enolase without influencing catalytic activity, points to a more structural role of AKT2/α-enolase interaction. Kinases may also act by organizing protein complex formation independent of their catalytic function [47]. Therefore, the association of AKT and several glycolytic enzymes might enable a tight regulation of the glycolytic cascade and formation of a metabolic aggregate with efficient channeling of substrates through the enzymatic chain similar to findings in plants [48] and other mammalian cells [49], [50].

**Figure 7:**
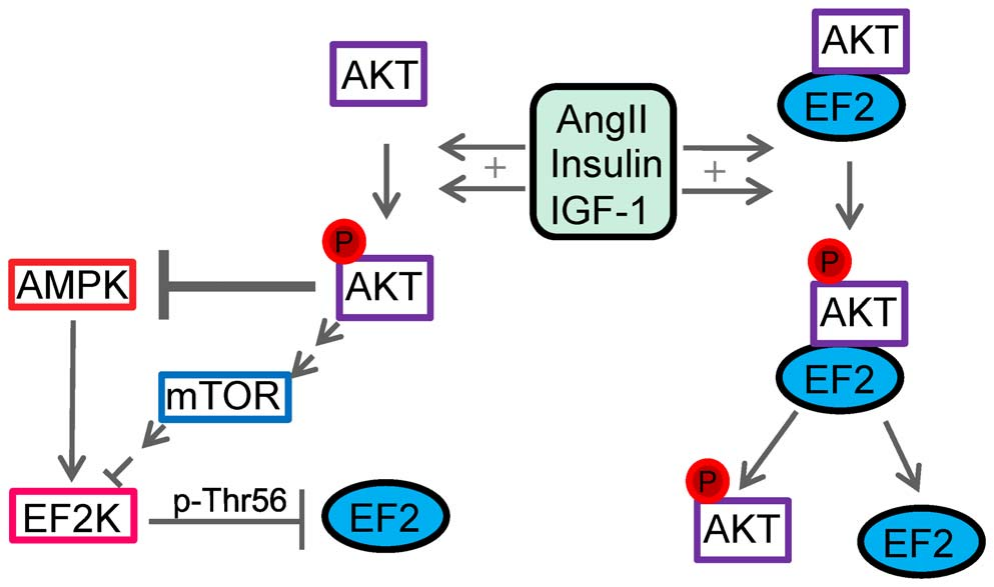
The AKT/EF2 connection. AKT is indirectly involved in EF2-activation via mTOR, AMPK and EF2K. Our data reveal a direct interaction of AKT2 and EF2, which is dynamically regulated upon angiotensin II, IGF-1 and insulin stimulation.

Elongation factor 2 (EF2) is a further newly discovered AKT2-interaction partner which may provide a direct link of AKT and cellular growth. This interaction was not specific for AKT2, because PLA performed with AKT1- and EF2-specific antibodies revealed that also AKT1 associates with EF2 in embryonic rat cardiac myocytes. EF2 modulates protein synthesis by transferring the ribosome during translation from 5′ to 3′. Translation can be easily disrupted by phosphorylation and thereby inhibition of EF2 on threonine 56 [51], a modification which is exerted by the highly specific EF2 kinase. Several links between AKT and elongation factor 2 were described in the literature (Fig. 7). It was reported that AKT on the one hand activates mTOR, which increases EF2-activity [52]. On the other hand, it inhibits AMPK which decreases EF2-activity [53]. Furthermore insulin and angiotensin II promote a PI3-kinase dependent dephosphorylation of EF2 [54], [55]. However, all of these regulatory effects were shown to act via EF2 kinase. Our data provide for the first time evidence of a direct interaction between AKT1/AKT2 and EF2, which suggests that AKT besides its indirect effects via mTOR, AMPK and EF2 kinase, may directly regulate EF2 by phosphorylation. Interestingly, the interaction of AKT with EF2 is subject to regulation. In serum deprived embryonic rat cardiac myocytes, angiotensin II induced AKT phosphorylation and led to a low level of AKT2/EF2 interaction. Inhibition of the PI3-kinase pathway leading also to AKT inhibition stabilized the AKT2/EF2 complex. Since similar results were obtained with insulin and IGF-1 we assume that activation of AKT may lead to activation of EF2 and the subsequent dissociation of AKT, providing access of EF2 to the ribosomes and stimulation of elongation. This in turn may explain, at least in part, the pro-hypertrophic function of AKT.

Taken together, TAP/MS based analysis reveals new AKT2 interacting proteins which support the function of AKT signaling in regulation of glucose metabolism and cell growth. Moreover, our study gives insight into the mechanism of AKT2-dependent signaling: Whereas this kinase forms rather stable complexes with chaperones and co-chaperones such as HSPs and Cdc37, the interaction with proteins executing specific cellular functions after AKT dependent phosphorylation appear to be rather unstable. In view of earlier findings that AKT substrates such as ASK1 or eNOS interact with a ternary complex consisting of AKT, HSP90 and Cdc37 our findings suggest that the interaction of AKT with chaperones to form a common interface for the interaction is a general principle of AKT-dependent signal transduction.

## Supporting Information

Figure S1 **To enable tandem affinity purification the AKT2-Tag construct was stably expressed in HEK293T cells.** The recombinant protein was under control of CAGGS promoter [56]. The vector for lentiviral infection was used as described before [57], [58]. For selection of infected cells puromycin was used (3 µg/ml).
(TIFF)

Figure S2 **Western blot analysis of TAP fractions of control TAP (A) and AKT2-Tag TAP (B).** A) Endogenous AKT from wild type cells can be detected in extract and flow through after the first purification step in almost equal amounts. A faint band is also visible in the wash fraction after Flag purification but not in Flag or Strep eluate. B) Cell extracts of AKT2-Tag cells show endogenous as well as recombinant AKT. While endogenous AKT is nearly completely lost in the first flow through, the recombinant protein can be detected in almost every fraction with accumulation in Flag and Strep eluate. C) Quantification of the TAP-efficacy. The purification yields approx. 18% of the initial AKT2-Tag protein.
(TIFF)

Figure S3 **Schematic overview of MBP- and MAP-procedure.** A) MBP: cell lysates of heavy and light labeled cells are mixed in a 1∶1 ratio upon cell lysis. After TAP and MS stably bound proteins show a shifted ratio, whereas transiently as well as unspecificly bound proteins show a 1∶1 ratio. B) MAP: TAP is performed with equal amounts of cell lysates from light and heavy labeled cells and afterwards the eluates are mixed in a 1∶1 ratio. After MS stable and transient complexes show shifted ratios whereas unspecificly bound proteins still show a ∼1∶1 ratio.
(TIFF)

Figure S4 **Western analysis of cell extracts which were used in the ENO1 assay.** To investigate the effect of CCT128930 on AKT substrates the anti-phospho AKT substrate (P-AKT-substrate) antibody was used. A) With insulin increased phosphorylation of substrates was achieved, while CCT-treatment led to an almost complete dephosphorylation of AKT substrates even in the presence of insulin. B) In contrast the phosphorylation of AKT at serine 473 was unaffected, since CCT128930 is an ATP-competitive AKT-inhibitor.
(TIFF)

Table S1 **Detailed overview over all detected proteins in 6 TAPs after in-gel digest.** The listed proteins were identified with more than three different peptides.
(PDF)

Protocol S1 **Preparation of embryonic rat cardiomyocytes.**
(PDF)

## References

[1] Hanks SK, Hunter T (1995) Protein kinases 6. The eukaryotic protein kinase superfamily: kinase (catalytic) domain structure and classification. FASEB J 9: 576–596. 7768349

[2] Inoki K, Li Y, Zhu T, Wu J, Guan KL (2002) TSC2 is phosphorylated and inhibited by Akt and suppresses mTOR signalling. Nat Cell Biol 4: 648–657 10.1038/ncb839. 12172553 10.1038/ncb839

[3] Zhou BP, Liao Y, Xia W, Spohn B, Lee MH, et al (2001) Cytoplasmic localization of p21Cip1/WAF1 by Akt-induced phosphorylation in HER-2/neu-overexpressing cells. Nat Cell Biol 3: 245–252 10.1038/35060032. 11231573 10.1038/35060032

[4] Datta SR, Dudek H, Tao X, Masters S, Fu H, et al (1997) Akt phosphorylation of BAD couples survival signals to the cell-intrinsic death machinery. Cell 91: 231–241. 9346240 10.1016/s0092-8674(00)80405-5

[5] Calera MR, Martinez C, Liu H, Jack AK, Birnbaum MJ, et al (1998) Insulin increases the association of Akt-2 with Glut4-containing vesicles. J Biol Chem 273: 7201–7204. 9516411 10.1074/jbc.273.13.7201

[6] Alessi DR, Andjelkovic M, Caudwell B, Cron P, Morrice N, et al (1996) Mechanism of activation of protein kinase B by insulin and IGF-1. EMBO J 15: 6541–6551. 8978681 PMC452479

[7] Alessi DR, James SR, Downes CP, Holmes AB, Gaffney PRJ, et al (1997) Characterization of a 3-phosphoinositide-dependent protein kinase which phosphorylates and activates protein kinase Bα. Current Biology 7: 261–269 10.1016/S0960-9822(06)00122-9. 9094314 10.1016/s0960-9822(06)00122-9

[8] Sarbassov DD, Guertin DA, Ali SM, Sabatini DM (2005) Phosphorylation and regulation of Akt/PKB by the rictor-mTOR complex. Science 307: 1098–1101 10.1126/science.1106148. 15718470 10.1126/science.1106148

[9] Cho H, Thorvaldsen JL, Chu Q, Feng F, Birnbaum MJ (2001) Akt1/PKBalpha is required for normal growth but dispensable for maintenance of glucose homeostasis in mice. J Biol Chem 276: 38349–38352 10.1074/jbc.C100462200. 11533044 10.1074/jbc.C100462200

[10] Tschopp O, Yang ZZ, Brodbeck D, Dummler BA, Hemmings-Mieszczak M, et al (2005) Essential role of protein kinase B gamma (PKB gamma/Akt3) in postnatal brain development but not in glucose homeostasis. Development 132: 2943–2954 10.1242/dev.01864. 15930105 10.1242/dev.01864

[11] Cho H, Mu J, Kim JK, Thorvaldsen JL, Chu Q, et al (2001) Insulin resistance and a diabetes mellitus-like syndrome in mice lacking the protein kinase Akt2 (PKB beta). Science 292: 1728–1731. 11387480 10.1126/science.292.5522.1728

[12] Gonzalez E, McGraw TE (2009) Insulin-modulated Akt subcellular localization determines Akt isoform-specific signaling. Proc Natl Acad Sci U S A 106: 7004–7009 10.1073/pnas.0901933106. 19372382 10.1073/pnas.0901933106 PMC2678468

[13] Gavin AC, Bösche M, Krause R, Grandi P, Marzioch M, et al (2002) Functional organization of the yeast proteome by systematic analysis of protein complexes. Nature 415: 141–147 10.1038/415141a. 11805826 10.1038/415141a

[14] Gavin AC, Superti-Furga G (2003) Protein complexes and proteome organization from yeast to man. Curr Opin Chem Biol 7: 21–27. 12547422 10.1016/s1367-5931(02)00007-8

[15] Basso AD, Solit DB, Chiosis G, Giri B, Tsichlis P, et al (2002) Akt forms an intracellular complex with heat shock protein 90 (Hsp90) and Cdc37 and is destabilized by inhibitors of Hsp90 function. J Biol Chem 277: 39858–39866 10.1074/jbc.M206322200. 12176997 10.1074/jbc.M206322200

[16] Sato S, Fujita N, Tsuruo T (2000) Modulation of Akt kinase activity by binding to Hsp90. Proc Natl Acad Sci U S A 97 10832–10837: 10995457. 10.1073/pnas.170276797 PMC27109 10995457

[17] Fontana J, Fulton D, Chen Y, Fairchild TA, McCabe TJ, et al (2002) Domain mapping studies reveal that the M domain of hsp90 serves as a molecular scaffold to regulate Akt-dependent phosphorylation of endothelial nitric oxide synthase and NO release. Circ Res 90: 866–873. 11988487 10.1161/01.res.0000016837.26733.be

[18] Shevchenko A, Wilm M, Vorm O, Mann M (1996) Mass spectrometric sequencing of proteins silver-stained polyacrylamide gels. Anal Chem 68: 850–858. 8779443 10.1021/ac950914h

[19] Reinartz M, Ding Z, Flögel U, Gödecke A, Schrader J (2008) Nitrosative Stress Leads to Protein Glutathiolation, Increased S-Nitrosation, and Up-regulation of Peroxiredoxins in the Heart. Journal of Biological Chemistry 283: 17440–17449. 18426799 10.1074/jbc.M800126200

[20] Vizcaíno JA, Côté RG, Csordas A, Dianes JA, Fabregat A, et al (2013) The Proteomics Identifications (PRIDE) database and associated tools: status in 2013. Nucleic Acids Research 41: D1063–D1069. 23203882 10.1093/nar/gks1262 PMC3531176

[21] Weser S, Gerlach M, Kwak DM-S, Czerwinska M, Gödecke A (2006) Detection of TAP-tagged proteins in Western blot, confocal laser scanning microscopy and FACS using the ZZ-domain. Journal of Biochemical and Biophysical Methods 68: 189–194 doi: 10.1016/j.jbbm.2006.06.002 16860393 10.1016/j.jbbm.2006.06.002

[22] Söderberg O, Gullberg M, Jarvius M, Ridderstråle K, Leuchowius KJ, et al (2006) Direct observation of individual endogenous protein complexes in situ by proximity ligation. Nat Methods 3: 995–1000 10.1038/nmeth947. 17072308 10.1038/nmeth947

[23] Yap TA, Walton MI, Hunter LJ, Valenti M, de Haven Brandon A, et al (2011) Preclinical Pharmacology, Antitumor Activity, and Development of Pharmacodynamic Markers for the Novel, Potent AKT Inhibitor CCT128930. Molecular Cancer Therapeutics 10: 360–371. 21191045 10.1158/1535-7163.MCT-10-0760 PMC4944842

[24] Oeljeklaus S, Reinartz BS, Wolf J, Wiese S, Tonillo J, et al (2012) Identification of Core Components and Transient Interactors of the Peroxisomal Importomer by Dual-Track Stable Isotope Labeling with Amino Acids in Cell Culture Analysis. J Proteome Res 11: 2567–2580 doi: 10.1021/pr3000333 22375831 10.1021/pr3000333

[25] Ong SE, Blagoev B, Kratchmarova I, Kristensen DB, Steen H, et al (2002) Stable isotope labeling by amino acids in cell culture, SILAC, as a simple and accurate approach to expression proteomics. Mol Cell Proteomics 1: 376–386. 12118079 10.1074/mcp.m200025-mcp200

[26] Wang X, Huang L (2008) Identifying dynamic interactors of protein complexes by quantitative mass spectrometry. Mol Cell Proteomics 7: 46–57 10.1074/mcp.M700261-MCP200. 17934176 10.1074/mcp.M700261-MCP200

[27] Tackett AJ, DeGrasse JA, Sekedat MD, Oeffinger M, Rout MP, et al (2005) I-DIRT, a general method for distinguishing between specific and nonspecific protein interactions. J Proteome Res 4: 1752–1756 10.1021/pr050225e. 16212429 10.1021/pr050225e

[28] Chen GI, Gingras AC (2007) Affinity-purification mass spectrometry (AP-MS) of serine/threonine phosphatases. Methods 42: 298–305. 17532517 10.1016/j.ymeth.2007.02.018

[29] Li Y (2011) The tandem affinity purification technology: an overview. Biotechnol Lett 33: 1487–1499 10.1007/s10529-011-0592-x. 21424840 10.1007/s10529-011-0592-x

[30] Zhang R, Luo D, Miao R, Bai L, Ge Q, et al (2005) Hsp90-Akt phosphorylates ASK1 and inhibits ASK1-mediated apoptosis. Oncogene 24: 3954–3963 10.1038/sj.onc.1208548. 15782121 10.1038/sj.onc.1208548

[31] Koren J III, Jinwal UK, Jin Y, O’Leary J, Jones JR, et al (2010) Facilitating Akt clearance via manipulation of Hsp70 activity and levels. J Biol Chem 285: 2498–2505 10.1074/jbc.M109.057208. 19889640 10.1074/jbc.M109.057208 PMC2807306

[32] Yamagishi N, Ueda T, Mori A, Saito Y, Hatayama T (2012) Decreased expression of endoplasmic reticulum chaperone GRP78 in liver of diabetic mice. Biochemical and Biophysical Research Communications 417: 364–370. 22155243 10.1016/j.bbrc.2011.11.118

[33] Pfaffenbach KT, Pong M, Morgan TE, Wang H, Ott K, et al.. (2012) GRP78/BiP is a novel downstream target of IGF-1 receptor mediated signaling. J Cell Physiol. 10.1002/jcp.24090. 10.1002/jcp.24090 PMC3421054 22422508

[34] Ni M, Zhang Y, Lee AS (2011) Beyond the endoplasmic reticulum: atypical GRP78 in cell viability, signaling and therapeutic targeting. Biochem J 434: 181–188. 21309747 10.1042/BJ20101569 PMC3353658

[35] Baba T, Kobayashi H, Kawasaki H, Mineki R, Naito H, et al (2010) Glyceraldehyde-3-phosphate dehydrogenase interacts with phosphorylated Akt resulting from increased blood glucose in rat cardiac muscle. FEBS Lett 584: 2796–2800 10.1016/j.febslet.2010.05.015. 20488185 10.1016/j.febslet.2010.05.015

[36] Huang Q, Lan F, Zheng Z, Xie F, Han J, et al (2011) Akt2 kinase suppresses glyceraldehyde-3-phosphate dehydrogenase (GAPDH)-mediated apoptosis in ovarian cancer cells via phosphorylating GAPDH at threonine 237 and decreasing its nuclear translocation. J Biol Chem 286: 42211–42220 10.1074/jbc.M111.296905. 21979951 10.1074/jbc.M111.296905 PMC3234938

[37] Colell A, Green DR, Ricci JE (2009) Novel roles for GAPDH in cell death and carcinogenesis. Cell Death Differ 16: 1573–1581. 19779498 10.1038/cdd.2009.137

[38] Altenberg B, Greulich KO (2004) Genes of glycolysis are ubiquitously overexpressed in 24 cancer classes. Genomics 84: 1014–1020 doi: 10.1016/j.ygeno.2004.08.010 15533718 10.1016/j.ygeno.2004.08.010

[39] Semenza GL, Jiang BH, Leung SW, Passantino R, Concordet JP, et al (1996) Hypoxia Response Elements in the Aldolase A, Enolase 1, and Lactate Dehydrogenase A Gene Promoters Contain Essential Binding Sites for Hypoxia-inducible Factor 1. Journal of Biological Chemistry 271: 32529–32537. 8955077 10.1074/jbc.271.51.32529

[40] Warburg O, Wind F, Negelein E (1927) The Metabolism of Tumors in the Body. J Gen Physiol 8: 519–530. 19872213 10.1085/jgp.8.6.519 PMC2140820

[41] Vander Heiden MG, Cantley LC, Thompson CB (2009) Understanding the Warburg Effect: The Metabolic Requirements of Cell Proliferation. Science 324: 1029–1033. 19460998 10.1126/science.1160809 PMC2849637

[42] Elstrom RL, Bauer DE, Buzzai M, Karnauskas R, Harris MH, et al (2004) Akt Stimulates Aerobic Glycolysis in Cancer Cells. Cancer Research 64: 3892–3899. 15172999 10.1158/0008-5472.CAN-03-2904

[43] Robey RB, Hay N (2009) Is Akt the “Warburg kinase”? Akt-energy metabolism interactions and oncogenesis. Seminars in Cancer Biology 19: 25–31. 19130886 10.1016/j.semcancer.2008.11.010 PMC2814453

[44] Gottlob K, Majewski N, Kennedy S, Kandel E, Robey RB, et al (2001) Inhibition of early apoptotic events by Akt/PKB is dependent on the first committed step of glycolysis and mitochondrial hexokinase. Genes & Development 15: 1406–1418. 11390360 10.1101/gad.889901 PMC312709

[45] Deprez J, Vertommen D, Alessi DR, Hue L, Rider MH (1997) Phosphorylation and Activation of Heart 6-Phosphofructo-2-kinase by Protein Kinase B and Other Protein Kinases of the Insulin Signaling Cascades. Journal of Biological Chemistry 272: 17269–17275. 9211863 10.1074/jbc.272.28.17269

[46] Brugarolas JB, Vazquez F, Reddy A, Sellers WR, Kaelin J (2003) TSC2 regulates VEGF through mTOR-dependent and -independent pathways. Cancer Cell 4: 147–158 doi: 10.1016/S1535-6108(03)00187-9 12957289 10.1016/s1535-6108(03)00187-9

[47] Patrucco E, Notte A, Barberis L, Selvetella G, Maffei A, et al (2004) PI3Kγ Modulates the Cardiac Response to Chronic Pressure Overload by Distinct Kinase-Dependent and -Independent Effects. Cell 118: 375–387. 15294162 10.1016/j.cell.2004.07.017

[48] Graham JWA, Williams TCR, Morgan M, Fernie AR, Ratcliffe RG, et al (2007) Glycolytic Enzymes Associate Dynamically with Mitochondria in Response to Respiratory Demand and Support Substrate Channeling. Plant Cell 19: 3723–3738. 17981998 10.1105/tpc.107.053371 PMC2174870

[49] Campanella ME, Chu H, Low PS (2005) Assembly and regulation of a glycolytic enzyme complex on the human erythrocyte membrane. PNAS 102: 2402–2407. 15701694 10.1073/pnas.0409741102 PMC549020

[50] Singh P, Salih M, Leddy JJ, Tuana BS (2004) The Muscle-specific Calmodulin-dependent Protein Kinase Assembles with the Glycolytic Enzyme Complex at the Sarcoplasmic Reticulum and Modulates the Activity of Glyceraldehyde-3-phosphate Dehydrogenase in a Ca2+/Calmodulin-dependent Manner. J Biol Chem 279: 35176–35182. 15199064 10.1074/jbc.M402282200

[51] Redpath NT, Price NT, Severinov KV, Proud CG (1993) Regulation of elongation factor-2 by multisite phosphorylation. European Journal of Biochemistry 213: 689–699 10.1111/j.1432-1033.1993.tb17809.x. 8386634 10.1111/j.1432-1033.1993.tb17809.x

[52] Wang X, Li W, Williams M, Terada N, Alessi DR, et al (2001) Regulation of elongation factor 2 kinase by p90RSK1 and p70 S6 kinase. EMBO J 20: 4370–4379 10.1093/emboj/20.16.4370. 11500364 10.1093/emboj/20.16.4370 PMC125559

[53] Horman S, Beauloye C, Vertommen D, Vanoverschelde JL, Hue L, et al (2003) Myocardial Ischemia and Increased Heart Work Modulate the Phosphorylation State of Eukaryotic Elongation Factor-2. Journal of Biological Chemistry 278: 41970–41976. 12920134 10.1074/jbc.M302403200

[54] Redpath NT, Foulstone EJ, Proud CG (1996) Regulation of translation elongation factor-2 by insulin via a rapamycin-sensitive signalling pathway. EMBO J 15: 2291–2297. 8641294 PMC450154

[55] Everett AD, Stoops TD, Nairn AC, Brautigan D (2001) Angiotensin II regulates phosphorylation of translation elongation factor-2 in cardiac myocytes. Am J Physiol Heart Circ Physiol 281: H161–H167. 11406481 10.1152/ajpheart.2001.281.1.H161

[56] Hitoshi N, Ken-ichi Y, Jun-ichi M (1991) Efficient selection for high-expression transfectants with a novel eukaryotic vector. Gene 108: 193–199. 1660837 10.1016/0378-1119(91)90434-d

[57] Leurs C, Jansen M, Pollok KE, Heinkelein M, Schmidt M, et al (2003) Comparison of Three Retroviral Vector Systems for Transduction of Nonobese Diabetic/Severe Combined Immunodeficiency Mice Repopulating Human CD34^+^ Cord Blood Cells. 14: 509–519. 10.1089/104303403764539305 12718762

[58] Drummond A, Ashton B, Buxton S, Cheung M, Cooper A, et al.. (2011) Geneious v5.4.

