# Supplementary figures and images for: Systematic Analysis Reveals Elongation Factor 2 and α-Enolase as Novel Interaction Partners of AKT2
Source: PLoS One. 2013 Jun 18;8(6):e66045. doi: 10.1371/journal.pone.0066045 (PMC3688836; doi:10.1371/journal.pone.0066045)

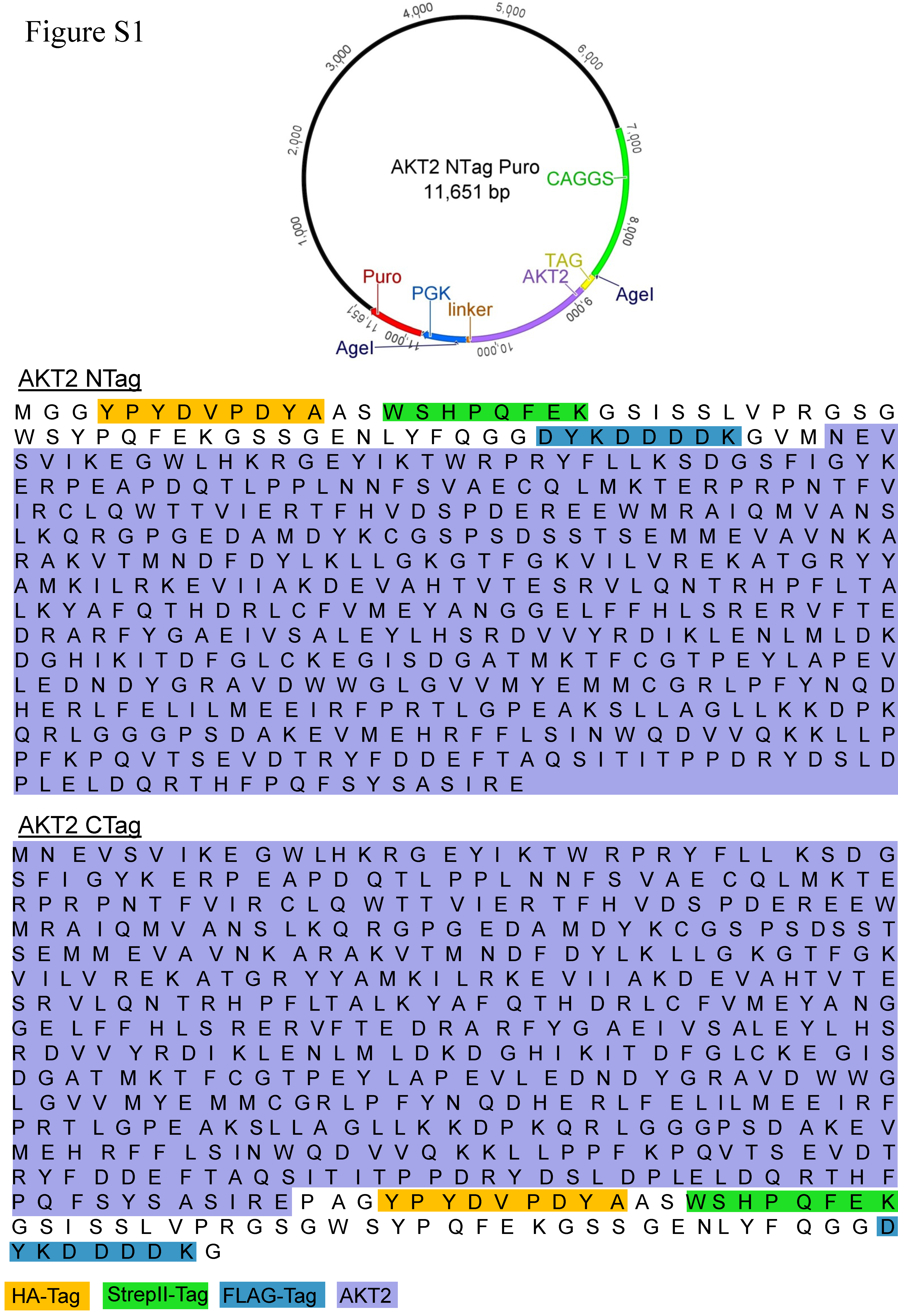

Supplement: Figure S1 — To enable tandem affinity purification the AKT2-Tag construct was stably expressed in HEK293T cells. The recombinant protein was under control of CAGGS promoter [56]. The vector for lentiviral infection was used as described before [57], [58]. For selection of infected cells puromycin was used (3 µg/ml). (TIFF) [file pone.0066045.s001.tiff]

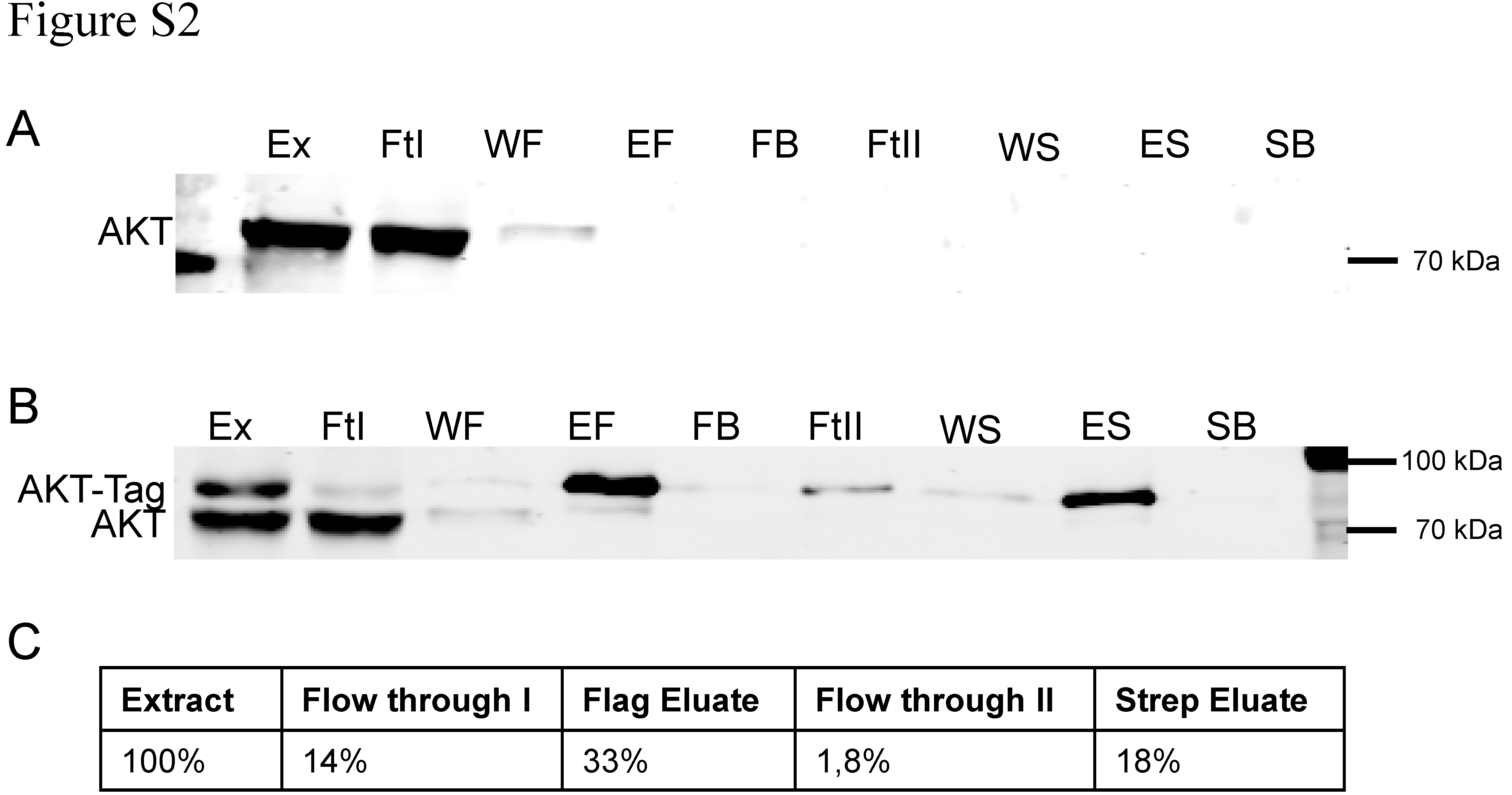

Supplement: Figure S2 — Western blot analysis of TAP fractions of control TAP (A) and AKT2-Tag TAP (B). A) Endogenous AKT from wild type cells can be detected in extract and flow through after the first purification step in almost equal amounts. A faint band is also visible in the wash fraction after Flag purification but not in Flag or Strep eluate. B) Cell extracts of AKT2-Tag cells show endogenous as well as recombinant AKT. While endogenous AKT is nearly completely lost in the first flow through, the recombinant protein can be detected in almost every fraction with accumulation in Flag and Strep eluate. C) Quantification of the TAP-efficacy. The purification yields approx. 18% of the initial AKT2-Tag protein. (TIFF) [file pone.0066045.s002.tiff]

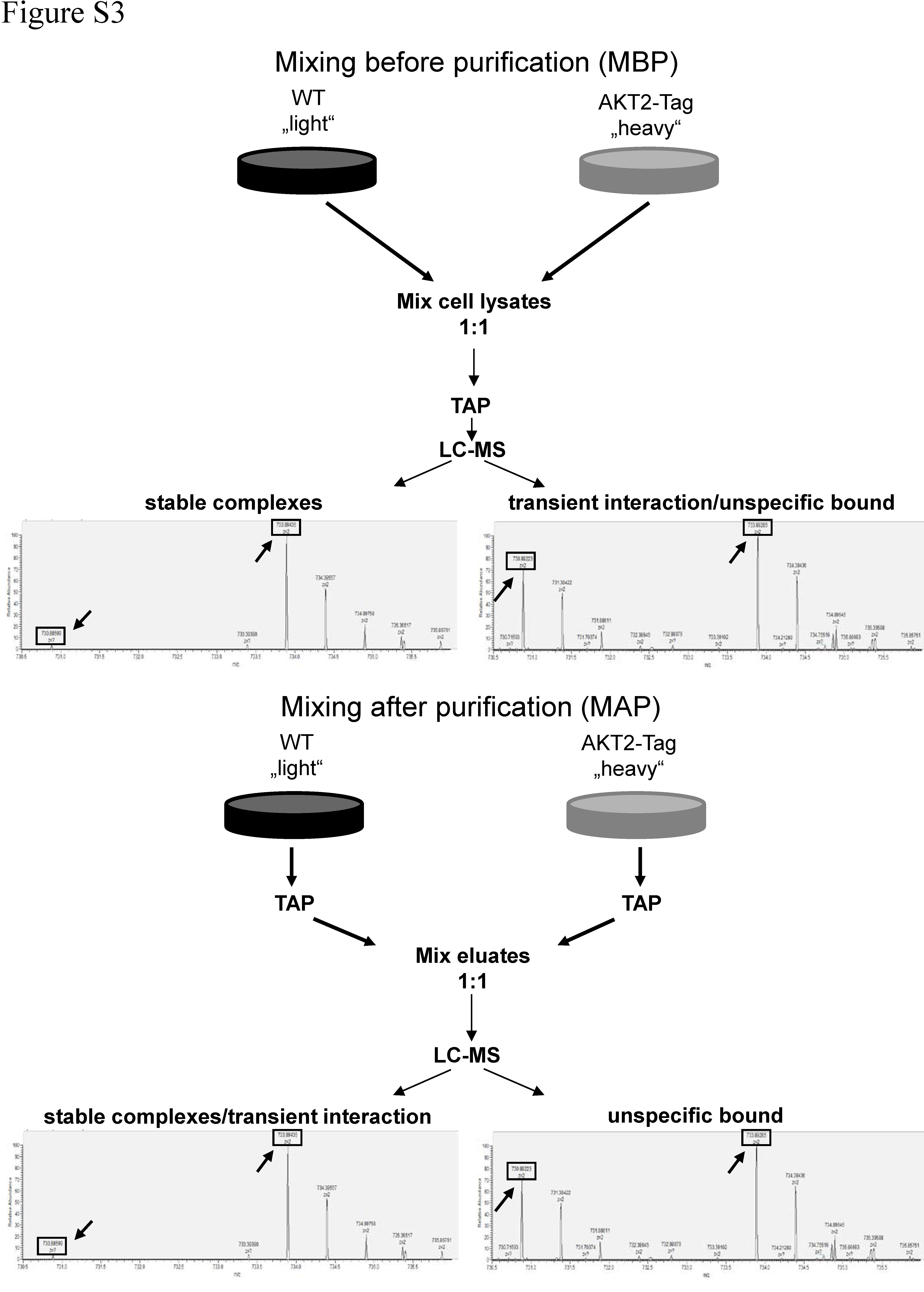

Supplement: Figure S3 — Schematic overview of MBP- and MAP-procedure. A) MBP: cell lysates of heavy and light labeled cells are mixed in a 1∶1 ratio upon cell lysis. After TAP and MS stably bound proteins show a shifted ratio, whereas transiently as well as unspecificly bound proteins show a 1∶1 ratio. B) MAP: TAP is performed with equal amounts of cell lysates from light and heavy labeled cells and afterwards the eluates are mixed in a 1∶1 ratio. After MS stable and transient complexes show shifted ratios whereas unspecificly bound proteins still show a ∼1∶1 ratio. (TIFF) [file pone.0066045.s003.tiff]

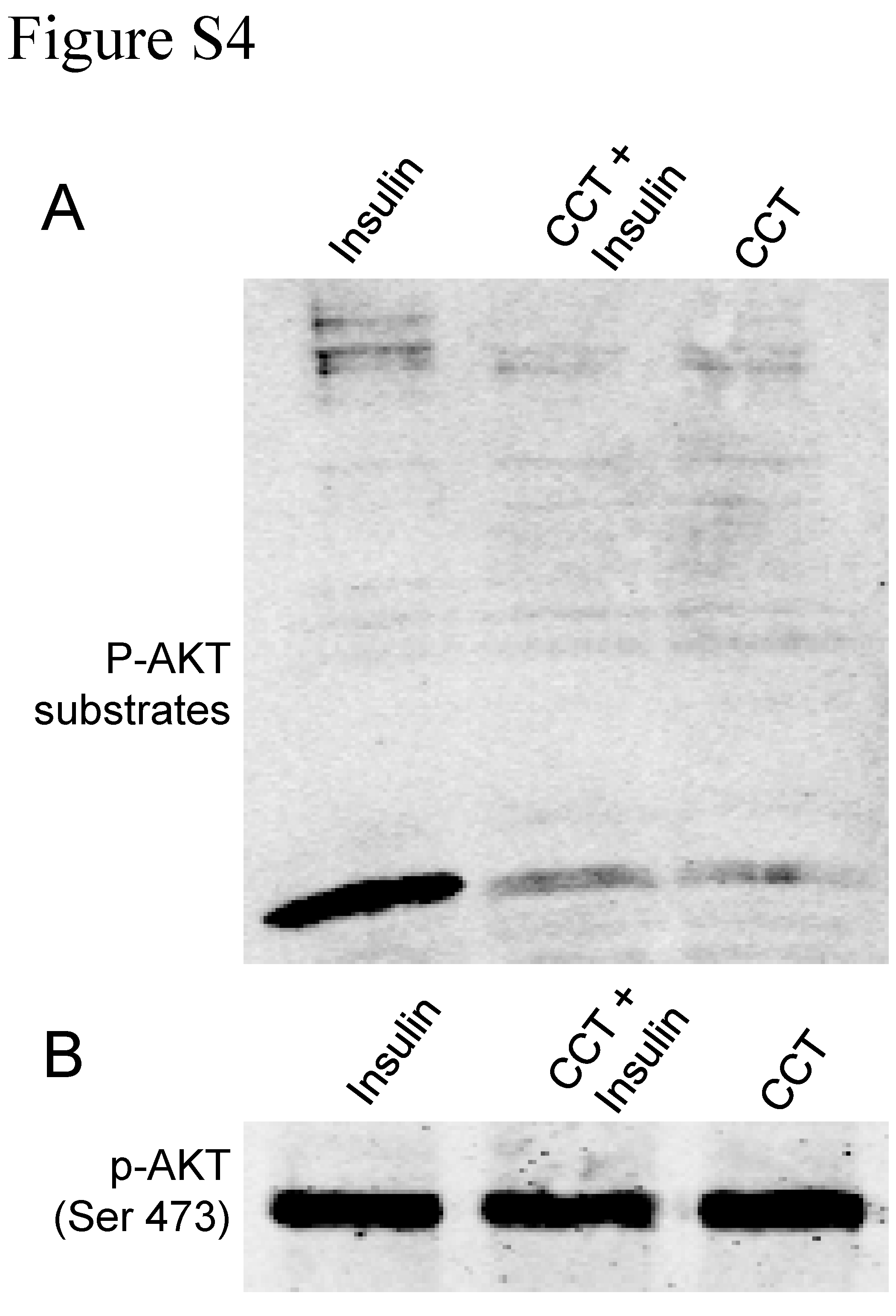

Supplement: Figure S4 — Western analysis of cell extracts which were used in the ENO1 assay. To investigate the effect of CCT128930 on AKT substrates the anti-phospho AKT substrate (P-AKT-substrate) antibody was used. A) With insulin increased phosphorylation of substrates was achieved, while CCT-treatment led to an almost complete dephosphorylation of AKT substrates even in the presence of insulin. B) In contrast the phosphorylation of AKT at serine 473 was unaffected, since CCT128930 is an ATP-competitive AKT-inhibitor. (TIFF) [file pone.0066045.s004.tiff]
