# Supplementary material for: Systematic Analysis Reveals Elongation Factor 2 and α-Enolase as Novel Interaction Partners of AKT2
Source: PLoS One. 2013 Jun 18;8(6):e66045. doi: 10.1371/journal.pone.0066045 (PMC3688836; doi:10.1371/journal.pone.0066045)
Supplement: Table S1 — Detailed overview over all detected proteins in 6 TAPs after in-gel digest. The listed proteins were identified with more than three different peptides. (PDF) [file pone.0066045.s005.pdf]

Table S1: Detailed overview over all detected proteins in 6 TAPs after in-gel digest. The listed proteins were identified with more than three different peptides.

AKT2-NTag I-VI	**amino acid sequence coverage**						**MW (kDa)**
	I	*II*	*III*	*IV*	*V*	*VI*	
**Proteins (accession number)**	AA SC%	AA SC%	AA SC%	AA SC%	AA SC%	AA SC%	
**Heat Shock Protein 90 kDa**							
Heat shock protein HSP 90-alpha (P07900)	9,3	5,7	14,8	19,9	27,4	28,4	84
Heat shock protein HSP 90-beta (P08238)	8,7	2,1	10,9	12,8		26,8	83
Cdc 37 (Q16543)	x	23,3	26,2	25,1	17,7	25,7	44
Endoplasmin (P14625)	x	x	5,2	x	32,8	38,6	92
**Heat Shock Protein 70 kDa**							70
Heat shock 70 kDa protein 1 (P08107)	x	26,7	14	37,8	42,6	37,1	70
Heat shock cognate 71 kDa protein (P11142)	28,2	30,8	-	35,3	46,6	47,8	70
Heat shock 70 kDa protein 1L (P34931)	6,2	5,8	5,9	16,4	24,6	18,3	70
Heat shock-related 70 kDa protein 2 (P54652)	8,4	7,4	-	10,5	7,8	10,5	70
78kDa glucose regulated protein (P11021)	36,5	x	39	39,1	43,3	40,2	72
Kinesin like protein (P52732)	x	x	51,3	x	x	x	119
Elongation Factor 2 (P13639)	x	x	15	x	46,4	51,4	95
Splicing factor U2AF 65 kDa subunit (P26368)	x	x	21,3	x	x	x	53
Elongation factor 1 alpha (P68104)	x	17,3	30,5	x	26	32,2	50
Tubulin							
Tubulin alpha-1A chain (Q71U36)	20,6	31	44,8	18	x	x	50
Tubulin beta-chain (P07437)	9,9	9,9	16	9,9	x	x	50
Tubulin beta-2A chain (Q13885)	15,5	24,5	36,2	15,7	x	x	50
Tubulin beta-2C chain (P68371)	7,4	10,8	16,9	7,4	x	x	50
Tubulin beta-1 chain (Q9H4B7)	6,2	6,4	6,4	6,4	x	x	50
Alpha-Enolase (P06733)	x	x	35,7	x	61,3	51,1	47
Creatine kinase B-type (P12277)	x	x	34,6	x	x	x	42
Methylosome protein 50 (Q9BQA1)	x	x	38,9	x	x	x	36
Glyceraldehyde-3-phosphate-dehydrogenase (P04406)	x	12,8	22,1	x	49,6	51,3	36
60S acidic ribosomal protein (P05388)	x	x	43,2	x	x	x	34
Putative heterogeneous nuclear ribonucleoprotein A1-like protein 3(P0C7M2)	x	x	28,9	x	x	x	34
40S ribosomal protein (P23396)	x	x	38,7	x	x	x	26
Cleavage and polyadenylation specifity factor su 5 (O43809)	x	x	41,4	x	x	x	26
BTB/POZ domain-containing protein KCTD5 (Q9NXV2)	x	x	35,9	x	x	x	26
Peroxiredoxin-1 (Q06830)	x	x	20,1	x	x	x	22
60S ribosomal protein (P62913)	x	x	37,6	x	x	x	20
Splicing factor, arginine/serine-rich 3 (P84103)	x	x	38,4	x	x	x	19
ANKRD26-like family C member 1A (Q6S8J3)	x	4,1	4,5	x	x	x	120
Actin, cytoplasmic (P60709)	x	x	x	x	13,3	13,3	41
Actin, aortic smooth muscle (P62736)	x	19,4	x	x	x	x	41
Ankyrin repeat domain-containing protein 1 (Q15327)	x	17,9	x	x	x	x	36
Triosephosphate isomerase (P60174)	x	16,1	x	x	x	x	26
ATP synthase subunit beta, mitochondrial (P06576)	x	15,3	x	x	x	x	56
Lactate dehydrogenase B chain (P07195)	x	25,8	x	x	17,1	19,5	36
Lactate dehydrogenase A chain (P00338)	x	11,7	x	x	x	x	36
Protein arginine N-methyltransferase 5 (O14744)	x	x	4,9	x	24,5	19,3	72
Nucleolin (P19338)	x	x	6,8	x	x	x	76
Importin (O95373)	x	x	8,3	x	x	x	119
Filamin A (P21333)	x	x	x	x	15,7	6,1	280
Filamin B (O75369)	x	x	x	x	x	3,7	278
Fatty acid synthase (P49327)	x	x	x	x	7	2,9	273
Acetyl CoA-Carboxylase (Q13085)	x	x	x	x	19,4	0,8	265
Clathrin heavy chain (Q00610)	x	x	x	x	30,3	22,75	191
Bifunctional aminoacyl-tRNA synthetase (P07814)	x	x	x	x	5,3	7,8	170
Eukaryotic translation initiation factor 3 subunit A (Q14152)	x	x	x	x	x	7,1	166
Structural maintenance of chromosomes protein 4 (Q9NTJ3)	x	x	x	x	x	8,5	147
Ubiquitin-like modifier –activating enzyme 1 (P22314)	x	x	x	x	22,9	28,7	117
Neutral alpha-glucosidase AB (Q14697)	x	x	x	x	27,1	36,9	106
Alpha-actinin-1 (P12814)	x	x	x	x	16,9	22,5	102
Malate dehydrogenase, cytoplasmic (P40925)	x	x	x	x	39,5	41,6	36
Malate dehydrogenase, mitochondrial (P40926)	x	x	x	x	31,9	43,2	35
Ezrin (P15311)	x	x	x	x	24,7	21,3	69
Protein disulfide-isomerase A4 (P13667)	x	x	x	x	29,8	28,2	72
Eukaryotic translation initiation factor 4B (P23588)	x	x	x	x	34	16,8	46
Phosphoglycerate kinase 1 (P00558)	x	x	x	x	34	34,3	44
