# Supplementary material for: Systematic Analysis Reveals Elongation Factor 2 and α-Enolase as Novel Interaction Partners of AKT2
Source: PLoS One. 2013 Jun 18;8(6):e66045. doi: 10.1371/journal.pone.0066045 (PMC3688836; doi:10.1371/journal.pone.0066045)
Supplement: Protocol S1 — Preparation of embryonic rat cardiomyocytes. (PDF) [file pone.0066045.s006.pdf]

Protocol S1

Preparation of embryonic rat cardiomyocytes

Adult pregnant Wistar rats were anesthetized with isofluoran and killed by cervical dislocation. Embryos were decapitated before preparing the hearts. All procedures were in accordance with the guidelines of the local Animal Care and Use Committee. Hearts were minced and cardiomyocytes were isolated by enzymatic dissociation with collagenase Type II (Gibco,1 mg/ml) and trypsin (Gibco, 3 mg/ml) in dissociation buffer (137 mM NaCl, 11 mM D-Glucose, 2,7 mM KCl, 12 mM $NaHCO_3$, 417 µM $NaH_2PO_4 \cdot H_2O$, 56 µM phenol red).Enzymatic dissociation was performed at 37°C for 30 min. Additional mechanical dissociation was done by repeated (every 5 min.) mixing and pipetting of the solution. After the dissociation procedure the solution was filtered through a 70µm cell strainer (BD Falcon) and the enzymatic reaction was stopped by adding ten volumes of culture medium standard DMEM (+ 20% FBS, 1% non-essential amino acids, 1% Penicillin/Streptomycin, 50µM β-Mercaptoethanol). Cells were pelleted by 5 min centrifugation at 800 rpm, resuspended in culture medium, plated at a density of 5x 10^5 cells/well and cultured at 37°C and 5% CO_2. On the next day cells were treated with mitomycin (10µg/ml) for 1 hour to prevent further proliferation of fibroblasts. The medium was changed every day.
